# Parents’ Lived Experiences With the COVID-19 Pandemic

**DOI:** 10.1177/1066480720969194

**Published:** 2020-11-10

**Authors:** Jo Lauren Weaver, Jacqueline M. Swank

**Affiliations:** 1 School of Human Development and Organizational Studies in Education, College of Education, University of Florida, Gainesville, FL, USA

**Keywords:** COVID-19, parents, parenting, family, children, pandemic

## Abstract

The COVID-19 pandemic has affected people across the globe. We explored 11 parents’ experiences with the pandemic and identified eight themes: (a) educational experience, (b) navigating roles and responsibilities, (c) recognizing privilege, (d) routine, (e) monitoring and communication about COVID, (f) vacillating emotions, (g) connection, and (h) meaningful experiences. We discuss the themes and implications for counseling.

On March 11, 2020, the World Health Organization (WHO, 2020) classified COVID-19 as a global pandemic, as the virus spread across more than 100 countries. In the United States, state and local governments issued stay-at-home orders affecting over 50% of all Americans (Mervosh et al., 2020). Schools and some employers transitioned to virtual settings. Nearly 90% of adults (*N* = 11,537) reported their lives had changed since COVID-19, with 44% indicating a major change (Pew Research Center, 2020).

## Effects of COVID-19 on Parents

The pandemic and quarantine resulted in multiple stressors for families. This included financial difficulties due to a 10.2% unemployment rate (U.S. Department of Labor, 2020), and sickness and fear of sickness. Among Australians (*N* = 1,536) in dual-earner homes, paid work time was slightly lower, and unpaid time (i.e., housework, childcare) was higher during quarantine (Craig & Churchill, 2020). Fathers also reported doing “much more” or “more” than their share of housework and childcare, thus reporting greater dissatisfaction with their work–family balance.

Parents also separated from support systems due to social distancing. Patrick et al. (2020) found 24% of parents reported a loss of childcare, and 35% reported struggling with managing childcare tasks (Pew Research Center, 2020). Researchers identify social support aiding in reduced caregiver distress and positively affecting parenting (McConnell et al., 2011). Parents may maintain a virtual connection with others; yet, they experience a loss of physical support. Parents may also be navigating multiple roles at home.

Some parents took on the teaching role as their children attended school virtually. Additionally, some worked from home, while others traveled to and from to work, risking exposure to the virus and tasked with finding childcare. Others served as caregivers to aging relatives and attempted to minimize virus exposure risk. Thus, parental roles and responsibilities increased as caregiver resources (e.g., family physical support) decreased.

Pandemic-related disruptions may also interfere with family engagement, including routines, rituals, and rules (Fiese et al., 2002). The stay-at-home orders caused shifts in family routines with children engaging in school virtually and parents working from home. Social distancing regulations affected routines, requiring families to make modifications. They also needed to maintain, but adapt, rituals to promote normalcy, which fosters resilience (Harrist et al., 2019). Moreover, parents had to establish new rules (e.g., schoolwork completion, social distancing). Thus, families experienced challenges requiring modifications in family engagement. While researchers have started investigating the effects of the pandemic on parents (e.g., Craig & Churchill, 2020), a need exists for exploring parents’ experiences with COVID-19. Our research question is “What are the lived experiences of parents during COVID-19?”

## Method

### Researchers

The two researchers identified as White females. One was a counselor educator, and one was a counselor education doctoral student. Both have worked with children and families with mental health concerns and amid crises. Both value the perspectives and experiences of parents and believe it is important to share their viewpoints related to COVID-19.

### Participants

Eleven parents with children aged 6–15 years old participated in the study. There were two males and nine females ranging in age from 33 to 49. One participant identified as Native American, and 10 identified as White, with one also identifying as Hispanic. Eight participants lived in a Southern U.S. state (Florida, Texas, and Kentucky), two lived in the Midwest (Ohio, Missouri), and one lived in the West (Montana). Regarding profession, three were counselors, three were counselor educators, one was a lawyer, one was a graduate assistant, one worked for the school board, one was a fitness instructor, and one worked as a consultant. Ten were working from home, and one was laid off. All participants’ children were attended school virtually.

### Procedure

Following the institutional review board approval, we recruited participants through Facebook parenting and pandemic support groups and a listserv for counselor educators, supervisors, and doctoral students (CESNET). We required participants to identify as a parent to one or more school-aged children (PreK–12). Individuals replied to the recruitment email, indicating a willingness to participate, and then we arranged the interview time and sent them a Zoom, a videoconference platform, link. The interviews occurred in April and May 2020. We also interviewed the children separately for a parallel project.

### Data Collection

The interview questions focused on parents’ experiences during the pandemic. This included discussing various facets of the parents’ lives. Parents also answered demographic questions (e.g., age, gender). Example questions included, “What do you think about what is going on in the world right now?” “What is different about your life right now?” We both interviewed parents and met periodically to verify consistency in our interview procedure.

### Data Analysis and Verification

We used a qualitative phenomenological data analysis approach (Moustakas, 1994). After reviewing and editing the transcriptions, prepared by Zoom, we sent them to participants to review for accuracy (member checking). None of the participants identified discrepancies nor wished to add to their interview. Separately, we analyzed the transcriptions to identify significant statements, group the statements into meaning units, delete similar statements, and identify emergent themes. Over a series of meetings, we obtained consensus on the themes.

## Findings

We identified eight themes. Additionally, we had five subthemes. The themes were (a) educational experience; (b) navigating roles and responsibilities with two subthemes, spousal relationship and letting go of expectations; (c) recognizing privilege; (d) routine with a subtheme of priorities; (e) monitoring and communication about COVID-19; (f) vacillating emotions; (g) connection with two subthemes, lost connection/ support, and changes in connections/relationships in the future; and (h) meaningful experiences.

### Educational Experience

Parents discussed the process of transitioning from in-person to virtual school. Trisha stated, “The schooling has been difficult…kids learn differently from their parents versus their teachers…something happens that’s not working right, or they don’t understand something, [and] they just break down, a meltdown.” Sharon reported,

He hates sitting…he thinks COVID had been the best thing…he’s getting everything he needs…talks to his friends all day long and he loves the online learning…he gets all his homework done by Tuesday.

Regarding the school structure, Pat remarked,

When they go to school…they’re at school to do school…the physical environment is conductive to that…Home…is where we play. We have a pretty small home…[We can’t] create a school space…very easily. [It is] distracted…blurring of boundaries.

Lisa stated,

She’s sort of expected to teach herself…Her teacher posts what you’re supposed to do this week, and she’ll do those things. They have synchronous Zoom meetings twice a week….[and] countless apps…[It is a] steep learning curve for me.

Concerning the online platform, Mark shared, “They’re doing Google Classroom, and at first it was really hard to navigate and figure out because I think they were struggling, and each teacher does it different too. There’s no uniformity.”

Some parents shared their concern for other students’ educational experiences during the COVID-19 pandemic. Janet commented,

The range of what kids go back to school with is going to be so different. There are kids who are…in front of the TV the entire day doing nothing, and…kids who are really hardcore learning.

Sandra remarked,

It’s just sad…I think a lot about schools and kids who really need to be in schools, and I worry about what’s happening with them…needing food and needing a safe space to be.

Additionally, parents discussed their children’s workload. Ken remarked, “[He has] a bunch of stuff he has to do, but I don’t know that he is aware of leaning much of anything…I think it’s just busy work.”

The parents discussed taking a different role in their children’s education. Dana shared,

[I am] an educator to my kid…I don’t have a curriculum…but I still feel a…responsibility to make sure he’s continuing to learn, even in a nontraditional way…I’m kind of enjoying [it] but…wish it was under different circumstances.

Janet remarked,

Alternating tasks and breaks has worked well…I’ve had to implement some ideas because…I think we got about 45 minutes of stuff to do a day…[I’m] supplementing what they can do all day.

Some parents described technological difficulties. Sandra stated,

The amount of involvement that I need to have to make sure that their schooling is happening is a lot more…[There are] a lot of glitches and bumps and 30 websites to log into with all different usernames and passwords.

Trisha commented,

If I just turn on the video…she wouldn’t pay attention…We’ve sat down to watch videos…These are long and boring and I don’t like them. [I tell her] let’s do this work and you can talk to me.

### Navigating Roles and Responsibilities

The participants reporting upholding multiple roles and responsibilities. Janet stated,

Like I’m running a relay race all day, and I’m really bad at it. Every time I look up to…see where I am, the races got longer…. My work has piled up…It’s exhausting and emotionally draining and physically draining.

Pat commented, “Trying to figure out my own balance…it’s just been very challenging…It’s just been survival mode…[to] get through the day.” Some parents expressed they were not given a break as their responsibilities increased. Mark discussed, “I felt like everybody got time off and I didn’t…I cannot do the same amount as I did before.” Other parents struggled with job loss. Trisha shared,

I was working before to not working now…Being a working person is a big part of who I am…[my] self-esteem, identity…that’s been my biggest struggle…It’s just part of self-fulfillment.

### Spousal Relationship

Parents shared different perceptions of the effects on their spousal relationship. Sharon remarked, “I feel really resentful and bitter towards my husband that his life goes on completely normally…not changed one bit…He goes to work.” Mark commented, “It’s important to be at your best for each other when you’re trapped in a house together.” Additionally, Trisha stated, “[We] partnered during this and communication…stability and strength…We split all of this.”

### Letting Go of Expectations

The participants reported letting go of expectations for themselves and their children. Regarding virtual school, Christy shared, “I’ve been trying to tell myself just let it go. Nobody is learning. When they all go back to school, whenever they do, everybody will be way behind the curve.” Dana stated,

Try to let go of the expectation of being the super homeschool mom, and being super work from home employee because it is an unreasonable expectation…Lower the expectations for yourself…for your kids.

Some parents discussed affording themselves grace. Trisha commented,

You don’t have to excel in all areas of your life during a global pandemic…Some days I feel like I’m winning for surviving…Give yourself grace….[I’m not doing] anything now with my kids that would be detrimental…. Relax…. and not judging others.

Sandra remarked, “Have grace with yourself and be forgiving…Give yourself a break.”

### Recognizing Privilege

Parents shared their experiences of privilege. Ken stated, “[It is] such a privileged answer really…Most of the things that we’ve experienced have been kind of inconveniences.” Some parents discussed their financial privilege. Lisa commented, “My husband is still working. We have a house. We have food…We’re not sacrificing a whole lot.” Mark remarked, “Our health and having stability,…we’re not worried about getting sick, and we’re not worried about being able to have food on the table and pay the mortgage.” Other parents viewed their privilege in not having to leave home. Pat stated, “I’m not scared of the world outside, but I also know that I have the privilege that I don’t have to go anywhere, and I’m fortunate that I don’t have to.”

### Routine

The participants discussed their changes in routine. Regarding parent routine, Marianne stated,

What my husband and I have kind of settled on…I take care of them [children] in the mornings…but then we rotate…. In the afternoons, I just go and it’s like four hours by yourself to try to get work done.

Janet shared, “I don’t have a commute anymore, so I’ve started working out almost every day. Similarly, with the time to do meal planning and stuff, we’re starting to eat healthier.” Regarding family routine, Sandra commented, “We establish[ed] some routines that kind of helped. We have morning meeting time…family check in time before the day starts…Sometimes we’ll do that around dinner [too].” Trisha remarked, “The whiteboard…here’s our checklist for today, just got to get through today.” Ken shared, “Get them [kids] involved in what you would normally do for chores. Teach them how to do laundry. Show them how to cook. Give them some life lessons.”

#### Priorities

Parents discussed reexamining their priorities. Dana reported, “[I’m trying to] figure out how to hold on to some of the slowness and reengaging at the same time, but not to go back to the frantic nature of the way I used to live.” Sandra stated,

Working mom guilt,…[they were] doing all these things…I felt guilty if I said no. Now, I don’t think I’m going to feel so compelled to do that. I hope it results in a sustainable slowing down.

Marianne commented,

[I] use my time more wisely…many hours in the day and so little gets done…I can have time for my family, instead of trying to do everything all at once and then everybody and everything suffering.

### Monitoring and Communication About COVID

The participants discussed filtering information about COVID-19 to their children. Janet remarked,

I’ve tried to shield a little bit of what’s going on and the severity…I don’t want to hide too much from her, but at the same time, she doesn’t need to be worrying, so trying to find a balance.

Christy commented,

Your kids watch you, and they feed off of you…They’re learning…by watching you. You are their safe place…You’re teaching them how to cope…how to handle crisis…how to deal with conflict and change.

Some parents discussed the media’s influence. Trisha stated,

Social media and just media and news can play a part in how people respond to a crisis…Have a balance, be informed. My motto was be cautious, not crazy…Fear really drives people.

Mark commented, “They have so much more knowledge of things that are really messed up in the world than I ever did as a kid, and I hate how desensitized they are to it.”

### Vacillating Emotions

Parents shared their emotional experience. Trisha commented, “A mix of emotions, and a roller coaster of one day I can be grateful and happy, and then the next day that I’m like, okay, this needs to be done, [but] this is overwhelming and too much.” Marianne stated,

It’s been a mixed bag. There are some days where I’m like this is awesome and I kind of hope it never ends…. Then, there are other days where…I feel like my moods have fluctuated more than they normally would.

Some parents discussed observing their children’s shifting emotions. Joan remarked, “He has days where he’s totally fine, then he has other days where I just get really worried…He tells me he’s fine.” Ken shared, “I’ve noticed my daughter already is experiencing this existential dread sometimes. She’s 8 years old, and sometimes she’ll cry for no reason.”

### Connection

The participants reported changes in connection. Regarding family connection, Janet commented,

The way that we’re interacting…level and quality of engagement…I’m hoping that doesn’t go away. We’re going to go back to spending less time together…[but] I want to be able to capture that…deeper level of engagement.

Mark added, “In some ways, we’ve had more interactions with family than we might even in normal times…. we’ve just been more intentional about it…[We] get together with 10 family members over zoom.” Sandra stated, “Probably out of this whole thing we’ve had a dozen family walks.” For some parents, this connection extended to other adults as well. Ken recalled, “[We have] gotten to know…neighbors.” Dana remarked,

We have a Friday night moms…. house party app…. we’ve met at a…parking lot…Back the cars up to one another and we sit in the trunk of the car and drink our coffee and talk…more connected with them.

Sandra commented, “Personally, I’m trying to keep in touch with friends via email, and we’re writing letters and doing virtual happy hours every once in a while.”

### Lost Connection/Support

Parents discussed their loss of connection and support. Marianne stated, “I’m not used to being so isolated and away from people. I crave human connection…Find[ing] new ways to cope has been difficult.” Christy shared, “Not having physical support from my immediate family…I feel the absence of that now and it’s weird because they’re so close; yet, we’re not seeing them right now.” Some parents discussed their children’s lost connections to peers and school. Janet commented, “They didn’t say goodbye to school and their friends the way they would normally. They missed out on that. I think they feel it…. Every night all the emotions kind of surface up.” Dana remarked, “I think the thing I miss the most is having my kids’ friends come over…all the group activities that I feel like have kept them so healthy for so long…team sports.”

#### Changes in connections/relationships in the future

Participants discussed how relationships might change post pandemic. Ken shared,

I worry about how this will change people. Relationships…the long term consequences of now we’re interacting on Zoom…My kids [have] more screen time than I ever could have imagined…changed us as a society and the politics behind everything.

Parents also discussed how this may change how children connect with others. Janet remarked, “We teach kids…about being inclusive and including people, and now you’re going to tell them to not touch them and wear a mask…It’s a mixed message.” Chole stated,

I definitely envision some things changed, but…I want my kids to…go to a playground…. [and] birthday parties. I want everything that we used to do…Go to our local pool and not worry…[have the] same social experiences.

### Meaningful Experiences

Parents shared their meaningful experiences during the pandemic. Regarding special events, Pat commented, “My older daughter did a virtual birthday party…with a friend from Pennsylvania, where we lived before. Normally, she wouldn’t have been able to go, but because it was virtual she was able to participate.” Sandra remarked,

[His] birthday,…his friends did a parade…They threw water balloons…They brought the cake and like set it down 10 feet from his feet…The neighbors were out on their steps and watching…[It was] very touching.

Some parents described how they were making daily experiences meaningful. Dana commented,

Focus on the present, being grateful for the things that we have and the relationships…finding fun things to do like building the tree house…a project that brings meaning and outlet for creativity.

Chole stated,

[Find] meaningful opportunities to engage in something that inspires your kid…to be creative and develop a sense of mastery and accomplishment.…. Don’t set your kid in front of the screen all day.

Mark shared, “[We are] finding enjoyment in the little things like literally walking through your neighborhood and looking at the wildlife.”

## Discussion

This is the first known study to explore parents’ lived experiences during the pandemic. We identified eight themes and five subthemes. The first theme focused on remote learning. Some parents reported positive aspects (e.g., not confined to a classroom). Yet, others described their children’s struggles. Some also voiced concern that teachers were assigning “busy work,” which researchers report can be detrimental academically (Snelling & Fingal, 2020). Parents also discussed their role, with some reporting being an educator to their children, as the virtual format did not facilitate learning. Others provided structure and supervision by allotting breaks, navigating the online platforms, and helping children stay focused.

In the second theme, parents reported taking on new roles with unique challenges (e.g., teaching). Additionally, one parent struggled with a loss of identity with being laid off. Regarding the subtheme, spousal relationship, researchers have reported the effects of the pandemic on work–family balance (e.g., Craig & Churchill, 2020), and an inverse correlation between marital satisfaction and levels of depression, anxiety, and stress (Wu et al., 2020). Hence, a need exists for marital satisfaction, as it relates to mental well-being. In the second subtheme, parents discussed letting go of their expectations. This shift underlies psychological flexibility or the ability to adapt and change to meet situational demands (Kashdan & Rottenberg, 2010). This flexibility can help parents cope with stress and uncertainty and ultimately buttress mental well-being in families during the pandemic (Coyne et al., 2020).

In the third theme, parents discussed financial privilege, their ability to work/stay home, and their health. Researchers identified individuals in certain work sectors (e.g., restaurants, transportation) being most affected by social distancing protocols (Vavra, 2020). Most parents in this study reported having professional occupations and not experiencing significant effects to their jobs. Additionally, most were White, perhaps reflecting the historically higher employment rate than those who identify as African American/Black and Latinx (Bureau of Labor Statistics, 2019). Responses could be echoing the preexisting labor/ wage gap in America, likely compounded by the pandemic. Parents also voiced staying physically well, which may highlight the racial health disparity emerging from COVID-19 (e.g., Millet et al., 2020).

The fourth theme focused on routines. For parental routine, some shared the benefits of more time at home and creating a new routine with their partner to balance work and childcare. For family routine, parents discussed needing more structure at home. This reflects routine as a type of family engagement (Fiese et al., 2002). Routines are important during uncertain times, as they create normalcy and can foster resilience (Harrist et al., 2019). Routines included morning meetings, to-do lists, and teaching “life lessons.” Through new routines, parents evaluated their priorities. They shared enjoying the slower pace and fewer activities, as it provided more family time. For some, this is a perceived benefit of the pandemic (Fegert et al., 2020).

The fifth theme involved parents censoring children’s exposure to the pandemic. Parents have a role in modeling coping responses, as highly distressed parental responses to a disaster can exacerbate negative child outcomes (Kerns et al., 2014). Hence, it is important for parents to be mindful of their reactions to the pandemic in front of their children. Researchers identified greater mental health concerns for individuals who had greater levels of media exposure in the aftermath of 9/11 (Galea et al., 2002). Today, children experience unprecedented access to the internet and media with 95% of adolescents owning a smartphone (Anderson & Jiang, 2018) and 42% of 0 to 8-year-olds having their own tablet (Rideout, 2017). Parents must decide how to monitor and filter their children’s exposure to the pandemic and how to educate them.

The sixth theme focused on the fluctuating emotional states. Researchers reported elevated levels of mental distress during the pandemic for parents (Patrick et al., 2020) and children (Golberstein et al., 2020). These highs and lows suggest reactions to the negative (e.g., social isolation) and positive (e.g., more family time) effects of the pandemic.

In the seventh theme, connections, parents described enhancing connection with their nuclear family, which is a possible benefit of the pandemic (Fegert et al., 2020). Researchers also reported an inverse relationship between parents’ intimacy level with their children and levels of depression, anxiety, and stress (Wu et al., 2020). Parents reported connecting with others on a deeper level, while also discussing loss of connection, as social distancing affects connecting with others outside the home. This loss can be detrimental (e.g., caregiver distress; McConnell et al., 2011); thus, connection and support remain important. Parents voiced concern for changes in future adult and child relationships. The effects of this unprecedented reliance on virtual connections are unknown. However, parents expressed hope that their children would connect with their peers as they did prior to the pandemic (e.g., in-person birthday parties).

The final theme was meaningful experiences and parents discussed having virtual birthday parties. Adapting existing rituals can engender normalcy and resilience (Harrist et al., 2019). Some parents focused on making daily experiences meaningful. Coyne et al. (2020) reported small things and creating meaning are important during the pandemic.

### Limitations and Recommendations for Future Research

Regarding limitations, there was a lack of participant diversity as the majority identified as White and female. Additionally, most participants were affiliated with the counseling profession and were able to work from home. Additionally, the majority were parents to elementary school-aged children who had access to virtual learning.

Future research may include replicating the study with a more diverse sample. Qualitatively, researchers may alter the protocol (e.g., interview parent dyads). Researchers may also conduct a follow-up study to explore the long-term effects of the pandemic. Furthermore, researchers may quantitatively examine the constructs identified through the themes.

### Implications

Regarding their children’s education, parents voiced satisfaction and frustration about virtual learning. Counselors may work with parents in navigating relationships and expressing concerns to school personnel (e.g., teachers). Additionally, counselors may encourage parents to consider creating a space in their home for school. Parents also reported the importance of establishing a routine. Family counselors could work with parents to establish this routine, which could create a sense of normalcy, and a positive learning environment. This may include establishing “periods” for activities, encompassing school subjects, and meaningful experiences for children (e.g., daily gratitude journaling; learning a new life skill). Moreover, counselors could help families brainstorm how to adapt special events (e.g., virtual birthday party).

Parents described loss of connection and feeling overwhelmed by more roles and responsibilities. Counselors may work with families to brainstorm strategies for connecting with others (e.g., virtual connections with friends, joining an online parent support group). This could also include virtual “playdates” for children, providing child socialization, and time for parental self-care. Furthermore, spouses may benefit from augmenting communication during this period of confinement. Couples counselors may introduce techniques (e.g., “I” statements) to enhance communication. Parents also described experiencing emotional highs and lows. Counselors working with parents could introduce stress reduction and mindfulness practices and endorse psychological flexibility and self-compassion. Mindful self-compassion includes promoting present-thinking, self-kindness, acknowledging our common humanity, and recognizing difficult thoughts and emotions without becoming attached (Neff, 2012).

Lastly, with unprecedented access to the news and social media, parents may struggle to filter the news their children receive about the pandemic. Counselors may suggest parents installing parental blocks/filters to devices, while monitoring what they consume on television and social media. Yet, perhaps most importantly, parents serve as models for their children in how to react to crises (Kerns et al., 2014). Hence, parents should be cognizant of their reactions, as their children will likely mirror their response. Counselors can also help parents discuss the pandemic with children in a developmentally appropriate manner.

## Conclusion

The unprecedented events of the pandemic warrant research to explore how families experience and navigate this experience. This study revealed that parents learning to adapt to this “new normal” experience unique challenges. Through resources and support, counselors can help parents navigate the pandemic and promote positive coping skills and family interactions.
